# Efficient Banknote Recognition Based on Selection of Discriminative Regions with One-Dimensional Visible-Light Line Sensor

**DOI:** 10.3390/s16030328

**Published:** 2016-03-04

**Authors:** Tuyen Danh Pham, Young Ho Park, Seung Yong Kwon, Kang Ryoung Park, Dae Sik Jeong, Sungsoo Yoon

**Affiliations:** 1 Division of Electronics and Electrical Engineering, Dongguk University, 30 Pildong-ro 1-gil, Jung-gu, Seoul 100-715, Korea; phamdanhtuyen@gmail.com (T.D.P.); fdsarew@hanafos.com (Y.H.P.); sbaru07@dgu.edu (S.Y.K.); jungsoft97@dongguk.edu (D.S.J.); 2 Kisan Electronics, Sungsoo 2-ga 3-dong, Sungdong-gu, Seoul 133-831, Korea; ssyoon@kisane.com

**Keywords:** banknote recognition, selection of distinguishable areas, one-dimensional visible-light line sensor, various types of banknote databases

## Abstract

Banknote papers are automatically recognized and classified in various machines, such as vending machines, automatic teller machines (ATM), and banknote-counting machines. Previous studies on automatic classification of banknotes have been based on the optical characteristics of banknote papers. On each banknote image, there are regions more distinguishable than others in terms of banknote types, sides, and directions. However, there has been little previous research on banknote recognition that has addressed the selection of distinguishable areas. To overcome this problem, we propose a method for recognizing banknotes by selecting more discriminative regions based on similarity mapping, using images captured by a one-dimensional visible light line sensor. Experimental results with various types of banknote databases show that our proposed method outperforms previous methods.

## 1. Introduction

The accurate and reliable recognition of banknotes plays an important role in the growing popularity of payment facilities such as ATMs and currency-counting machines. There have been many studies on this classification functionality that have been based on the optical characteristics of banknotes. Most studies on classification of banknotes by denomination (e.g., $1, $5, $10, *etc*.) have been based on images of banknotes captured by visible-light sensors. In general, a banknote can appear in four directions on two sides, *i.e.*, the forward and reverse images of the front and back sides, and the captured images of these input directions are used in recognition of banknotes.

Previous studies using visible-light images of banknotes can be divided into those that used whole banknote images for recognition [1,2,3,4,5,6] and those that used certain regions of banknote images [7,8,9,10,11,12]. Wu *et al.* [1] proposed a banknote orientation recognition method that uses the average brightness of eight uniform rectangles on a banknote image as the input of the classifier using a three-layer back-propagation (BP) network. However, their experiments only focused on orientation recognition of one type of Chinese banknote—the Renminbi (RMB) 100 Yuan note. A Chinese banknote recognition method using a three-layer neural network (NN) was proposed by Zhang *et al.* [2]. This method uses linear transforms of gray images to reduce the effect of noise and uses the edge characteristics of the transformed image as the input vectors to the NN classifier. This method was applied to Sri Lankan banknote recognition in [3]. A BP network was used as the classifier in research by Gai *et al.* [4]. In this research, recognition features were extracted by applying generalized Gaussian density (GGD) to the capture of the statistical characteristics of quaternion wavelet transform (QWT) coefficients on banknote images. To recognize multiple banknotes, Hassanpour and Farahabadi considered the texture characteristics of paper currencies as a random process and used hidden Markov model (HMM) for classification [6]. The Indian banknote recognition method proposed by Sharma *et al.* [5] uses a local binary pattern (LBP) operator to extract features from banknote images and classifies banknote types using Euclidean distances with template images.

In studies concerning regions of banknotes, not all the image data that a banknote provides have been used for recognition; only certain areas on banknote images have been selected and used. This helps to reduce the amount of input data required and puts the focus on regions of banknotes that have high degrees of discrimination. A Bangladeshi banknote recognition system was proposed using axis symmetric masks to select regions of banknote images before feeding information into a multilayer perceptron network to reduce the network size and adapt it to banknote flipping [7]. Axis-symmetrical masks were also applied to the neuro-recognition system proposed by Takeda and Nishikage [8] for analysis of Euro currency using two image sensors. Takeda *et al.* also proposed a mask optimization technique using a genetic algorithm (GA) [10] that could be used to select good masks using the sum of the pixels uncovered by masks, called the slab value [9], as the input to the recognition neural network. Component-based banknote recognition with speeded-up robust features (SURF) was proposed by Hasanuzzaman *et al.* [11]. In this method, components that provide specific information about banknotes, such as denomination numbers, portraits, and building textures, are cropped and considered to be reference regions. In the multi-currency classification method proposed by Youn *et al.* [12], multi-template correlation matching is used to determine the discriminant areas of each banknote that are highly correlated among banknotes of the same types and poorly correlated among those of different types.

Another approach to extracting classification features involves using statistical procedures such as principal component analysis (PCA) [13,14,15,16] or canonical analysis (CA) [17] to reduce the size of the feature vector. In research using learning vector quantization (LVQ) as the classifier, input feature vectors have been extracted by PCA from banknote data acquired by various type of sensors, such as sensors of various wavelengths [13], or point and line sensors [14]. The banknote recognition method proposed by Rong *et al.* [15] for a rail transit automatic fare collection system employs PCA to extract features and build matching templates from banknote data acquired by various sensors, such as magnetic, ultraviolet (UV) fluorescence and infrared (IR) sensors. In the hierarchical recognition method proposed by Park *et al.* [16], United States dollar (USD) banknotes were classified by front or back size and their forward or backward directions using a support vector machine (SVM) and then recognized by denomination ($1, $2, $5, *etc*.) using a K-mean algorithm based on the PCA features extracted from sub-sampled banknote images. In research by Choi *et al.* [17], CA was used for size reduction and to increase the discriminating power of features extracted from Korean won images using wavelet transform.

There have also been studies combining both of the above feature extraction approaches. The Indian currency recognition method proposed by Vishnu and Omman [18] selects five regions of interest (ROI): the textures of center numerals, shapes, Reserve Bank of India (RBI) seals, latent images, and micro letters on scanned banknote images. PCA is subsequently used for dimensionality reduction of the extracted features. Finally, the recognition results are validated using a classifier implemented with WEKA software [18]. Texture-based analysis is also used in the Indian banknote recognition method proposed by Verma *et al.* [19]. In this method, linear discriminant analysis (LDA) is applied to ROIs containing textures on banknote images for feature reduction, and SVM is applied for classification. Here, the ROI selection is conducted with the help of a set of external tools called Mazda. In the smartphone-based US banknote recognition system proposed by Grijalva *et al.* [20], regions of interest are located in the right parts of banknote images. From these regions, weight vectors are extracted using PCA and are compared with those of a training set using the Mahalanobis distance to determine the denomination of an input banknote. Although ROIs were defined in [20], it is uncertain whether the selected areas on banknote images are indeed those with highest discriminating power for recognition purposes.

To overcome these limitations, we propose a banknote recognition method that uses a combination of both of the feature extraction approaches mentioned above. From the sub-sampled banknote images, we select areas that have high degrees of similarity from among banknotes in the same class and high degrees of difference among those in different classes. The discriminant features are then extracted from the selected data using PCA, and the banknote type is determined by the classifier based on K-means algorithm. Our method is novel in the following respects:

(1) Using the sub-sampled banknote region from the captured image, the local areas that have high discriminating power are selected using a similarity map. This map is obtained based on the ratio of correlation map values, considering between-class and in-class dissimilarities among banknote images.
(2) Optimally reduced features for recognition are obtained from the selected local areas based on PCA, which reduces both the noise components and the processing time.
(3) The performance of our method has been measured using both normal circulated banknotes and test notes, and the effectiveness of our method has been confirmed in the harsh testing environment of banknote recognition.
(4) Through experiments with various types of banknotes—US dollars (USD), South African rand (ZAR), Angolan kwanza (AOA) and Malawian kwacha (MWK)—we have confirmed that our method can be applied irrespective of the type of banknote.

Table 1 presents a comparison between our research and previous studies. The remainder of this paper is organized as follows: Section 2 presents the details of the proposed banknote recognition method. Experimental results are presented in Section 3, and conclusions drawn from the results are presented in Section 4.

## 2. Proposed Methods

### 2.1. Overview of the Proposed System

**Figure 1:**
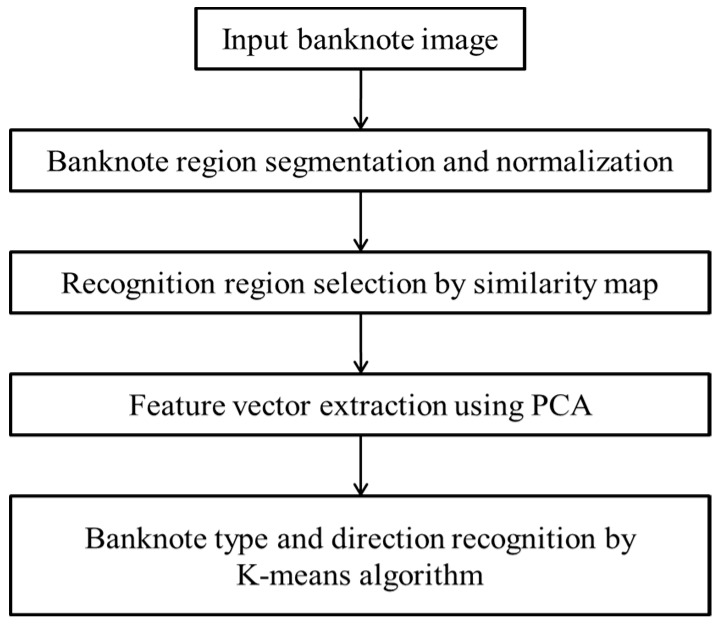
Flowchart of proposed method.

Figure 1 is an overview of the proposed banknote recognition system. The pre-processing step for acquired banknote images is as follows. A banknote region is segmented from the input image and sub-sampled to a size of 64 × 12 pixels to reduce the processing time. In the second step, from the sub-sampled banknote image, the recognition region with the high discriminating power is selected using a similarity map. Consequently, the optimally reduced feature vector is extracted from the data selected with the similarity map using PCA. Finally, the banknote type and the direction of the input image are determined using a K-means algorithm based on the PCA features.

### 2.2. Acquisition of Banknote Image, Region Segmentation and Normalization

**Figure 2:**
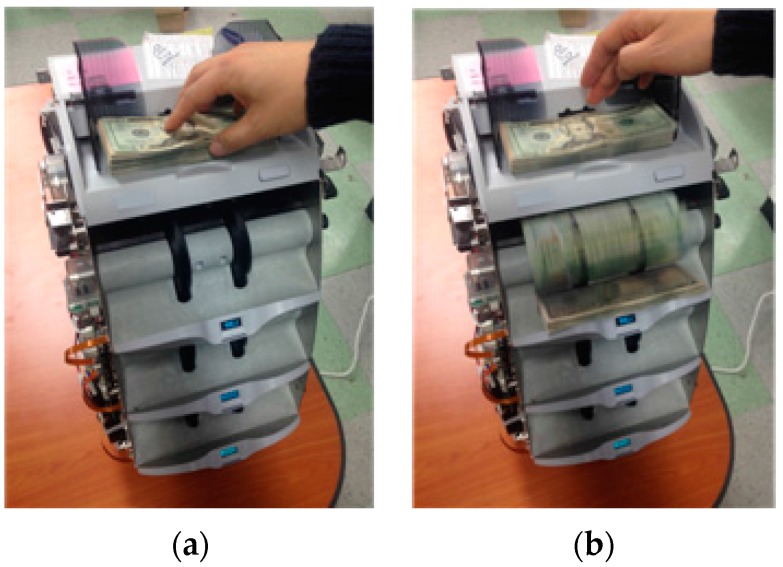
Examples of the set-up of our research: (**a**) Input banknotes. (**b**) Acquisition of image data.

In this study, we used a commercial banknote-counting machine [21]. Figure 2 shows the set-up of our research. As shown in Figure 2a, if we input the banknotes into the banknote-counting machine, the image data of each banknote can be automatically acquired as shown in Figure 2b. Because of the limitations of the size and cost of the counting machine, a conventional two-dimensional (area) image sensor is not used. One line image is captured at each trigger time as the input banknote is moving through the roller device inside the counting machine at a high speed and is being illuminated by a light-emitting diode (LED). The line sensor has a resolution of 1584 pixels and is triggered to capture 464 line images for each moving input banknote. A 1584 × 464 pixel banknote image of visible light is acquired by concatenating the line images.

**Figure 3:**
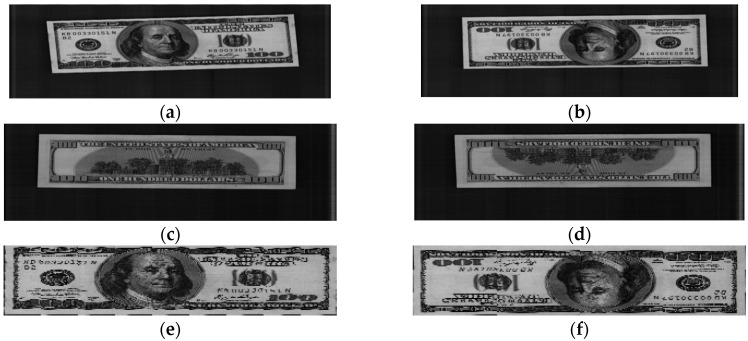

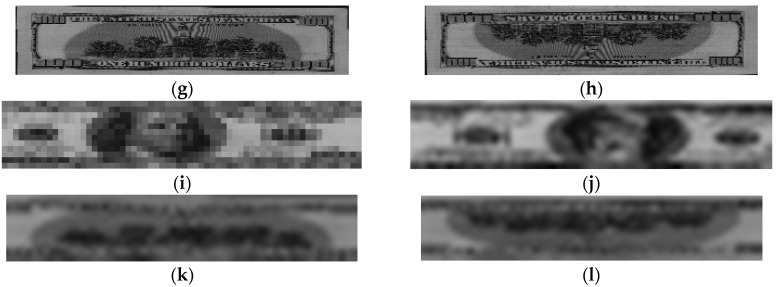
Examples of input images for four banknote directions: (**a**) A direction; (**b**) B direction; (**c**) C direction; (**d**) D direction; (**e**–**h**) Corresponding banknote areas segmented from the images in (**a**–**d**), respectively; (**i**–**l**) Corresponding 64 × 12-pixel sub-sampled images of the banknote area segmented images in (**e**–**h**), respectively.

When entering the recognition system, a banknote can be exposed in one of the following four directions: the front side in the forward direction (the “A direction”) or backward direction (the “B direction”), the back side in the forward direction (the “C direction”) or backward direction (the “D direction”). In this study, we classified banknotes in terms of type (e.g., $1, $5, $10) and direction. Therefore, there are four classes corresponding to four directions for each type of banknote. To address the problems of displacement and rotation of the banknote area in the captured image [16], we use the commercial corner detection algorithm built into the counting machine to locate the banknote area and exclude the background area from the captured banknote image, as shown in Figure 3. The segmented banknote images are then sub-sampled to have the same size of 64 × 12 pixels to reduce the effect of noise and redundant data and to increase the processing speed. Examples of original banknote images, corresponding banknote area segmented images, and sub-sampled images are shown in Figure 3.

### 2.3. Similarity Map

In a sub-sampled banknote image, there are areas that are mostly similar regardless of the banknote type and areas that are more distinguishable among different types of banknotes. To properly select the highly discriminative regions for recognition of banknote type, we propose a method based on the calculation of the ratio of the similarity among sub-sampled banknote images in the different classes and in the same class. This method results in a 64 × 12-pixel binary mask called a similarity map that can be obtained from a training data set using the following procedure.

**Figure 4:**
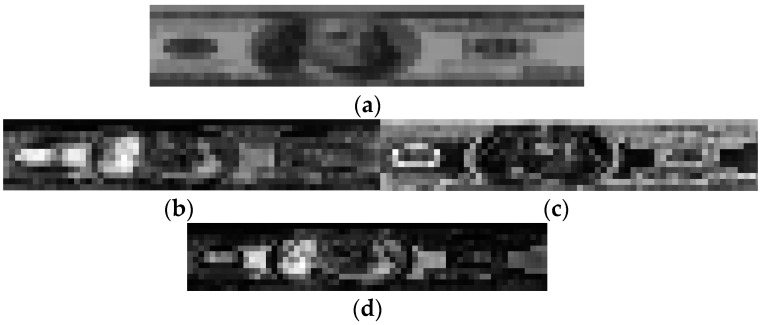
Examples of correlation maps and similarity map of front-forward recent US$100 banknote image: (**a**) Reference image; (**b**) Between-class correlation map; (**c**) In-class correlation map; (**d**) Similarity map.

First, we generate a reference banknote image for each class by averaging all the banknote images belonging to the same class. An example of a reference image of a recent US $100 banknote in the front-forward direction is shown in Figure 4a. Based on the reference images generated, we calculate the correlation maps for each input banknote with respect to the class to which it belongs in the training set using the following formulas:

(1) $$M(i,j)=\left|I^{\prime }(i,j)-R^{\prime }(i,j)\right|$$

with

(2) $$I^{\prime }(i,j)=\frac{I(i,j)-\mu_{I}}{\sigma_{I}}$$

(3) $$R^{\prime }(i,j)=\frac{R(i,j)-\mu_{R}}{\sigma_{R}}$$

where *I*(*i, j*) and *R*(*i, j*) are the gray-scale values of the pixel at position (*i, j*) of the input image and reference image, respectively; *µ_I_* and *σ_I_* are the mean and standard deviation values of the input image; and *µ_R_* and *σ_R_* are the mean and standard deviation values of the reference image. If the input banknote image and reference image are in the same class, the correlation map is defined as an in-class correlation map, denoted by *M_IC_*(*i, j*); otherwise, it is defined as a between-class correlation map, denoted by *M_BC_*(*i, j*). By taking the average images of all the in-class and between-class correlation maps of all the training banknote images in each class, we obtain the in-class and between-class correlation maps of each class, denoted by ${\bar{M}}_{IC}(i,j)$ and ${\bar{M}}_{BC}(i,j)$, respectively. Examples of visualized between-class and in-class correlation maps of recent US$100 banknotes in the front-forward direction are shown in Figure 4b,c.

In the next step, the similarity map of each class is calculated by determining the pixel-wise ratio of between-class and in-class correlation maps, using Equation (4). If a pixel has an in-class map value equal to zero, its similarity map value is assigned the maximum value among those of the other calculated pixels. An example of a visualized similarity map for a front-forward US$100 banknote image is shown in Figure 4d.

(4) $$S(i,j)=\frac{{\bar{M}}_{BC}(i,j)}{{\bar{M}}_{IC}(i,j)}$$

Using Equation (4), we can determine the areas where the dissimilarity of banknotes from the different classes is higher than that of the same class. These areas are the regions that have high discriminating power for banknote images and are represented by the bright pixels in the similarity map, scaled to the gray values, as shown in Figure 4.

**Figure 5:**
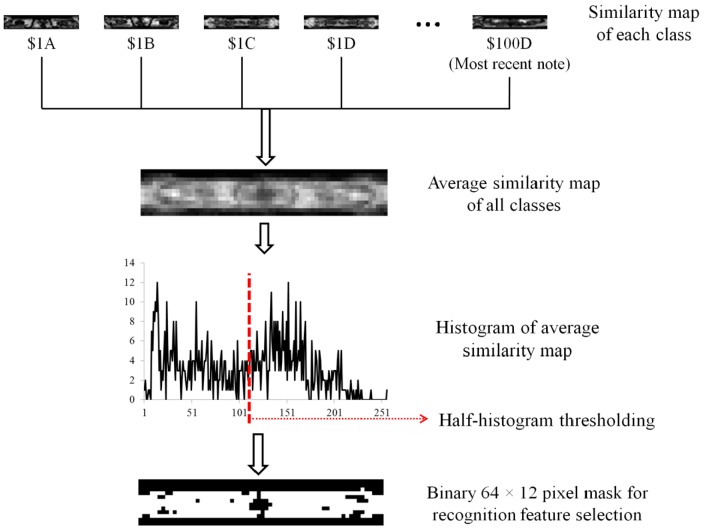
Example of average similarity map obtained from similarity maps of all USD classes and binary mask obtained from similarity map for feature selection.

Finally, we average the similarity maps of all the banknote classes to obtain the final similarity map. To select the banknote areas corresponding to the bright pixels on the similarity map, we use the thresholding method, so that the histogram of the similarity map is divided by half, the higher map values are assigned “1”, and the lower values are assigned “0”. The resulting binary similarity map image is considered to be a mask for selecting the pixels at white mask positions (corresponding to bright similarity map values) on the sub-sampled banknote image. These are used in recognition of the banknote type. The number of pixels selected for use in recognition is roughly half of the original sub-sampled image (approximately (64 × 12)/2 = 384 pixels). This procedure and examples of each intermediate stage are illustrated in Figure 5.

### 2.4. Feature Extraction by PCA and Classification by K-Means Algorithm

#### 2.4.1. PCA Method and PCA-Based K-Means Algorithm

To further reduce the number of dimensions of the input vector, we apply the PCA method to banknote data selected using the similarity map in the previous step. PCA is a statistical procedure for representing the data in a lower-dimensional space by projecting original data onto the eigenvectors corresponding to the largest eigenvalues of the covariance matrix. The procedure for conducting PCA in our research is similar to that used in the eigenface method [20,22]. First, we calculate the covariance matrix of the mean-subtracted training data using the following formula:

(5) $$C=\frac{1}{N}XX^{T}$$

where $X={\left[\begin{array}{cccc} \left(x_{1}-\mu \right) & \left(x_{2}-\mu \right) & ... & (x_{N}-\mu \end{array})\right]}^{T}$ is the *µ* mean-subtracted vector from the input data *x_i_* (*i* = 1,…,*N*) and *N* is the number of original data values.

From the covariance matrix C, we calculate the eigenvalues and eigenvectors of C. The N eigenvalues [*λ*_1_, *λ*_2_, …, *λ_N_*] are sorted in descending order, and their corresponding eigenvectors are arranged row by row to form the matrix V, as follows:

(6) $$V=\left[\begin{array}{c} v_{1}\\ v_{2}\\ ...\\ v_{N} \end{array}\right]$$

where v*_i_* is the eigenvector corresponding to eigenvalue *λ_i_*, *i* = 1, …, *N*. Matrix V is of size *N* × *N*. If we need to reduce the input data size to *M* smaller than *N*, the projection of X onto the first *M* eigenvectors’ directions is conducted, and results in Y, which consists of the coefficients of the principal components of X, as shown in the following equation:

(7) $$Y=V_{M}X=\left[\begin{array}{c} v_{1}\\ v_{2}\\ ...\\ v_{M} \end{array}\right]\left[\begin{array}{c} \left(x_{1}-\mu \right)\\ \left(x_{2}-\mu \right)\\ ...\\ \left(x_{N}-\mu \right) \end{array}\right]$$

The sizes of V*_M_*, X, and Y are *M* × *N*, *N* × 1, and *M* × 1, respectively. As a result, the banknote data are represented by the PCA coefficients in lower dimensionality, and we use these coefficients as inputs to the classifier in the next step.

The features extracted by PCA are used for classification of the banknote type and direction. The number of classes is predefined as the number of denominations to be recognized multiplied by four directions. When a banknote is input into the system, its recognition features are extracted, and the type and direction are determined based on the Euclidean distance to the class centers (vectors), which are obtained using a K-means clustering algorithm [23]. For example, in the case of USD, the number of classes is 68, which equals 17 types of banknotes ×4 directions (A, B, C, and D). Therefore, there are 68 class centers in the training result for USD. Using the extracted features of an input banknote, the distances between this PCA feature vector and the 68 center vectors are calculated, and the banknote is determined to belong to the class with the nearest center to the banknote’s feature (the nearest centroid classifier [24]).

#### 2.4.2. Determination of Number of PCA Dimensions used for Feature Extraction Method

A typical nearest-centroid-based classifier uses the shortest distance between the input vector and the center vectors as the class assignment criterion. However, there are cases in which the class assignment is not certain, e.g., when the input vector is located at nearly the central position between two class centers. In such a case, the difference between the shortest and second-shortest distances of input vector to the class centers is small. In this study, we considered both the shortest distance, referred to as the 1st distance, and the second-shortest distance, referred to as the 2nd distance, to evaluate the certainty and effectiveness of the classification results.

**Figure 6:**
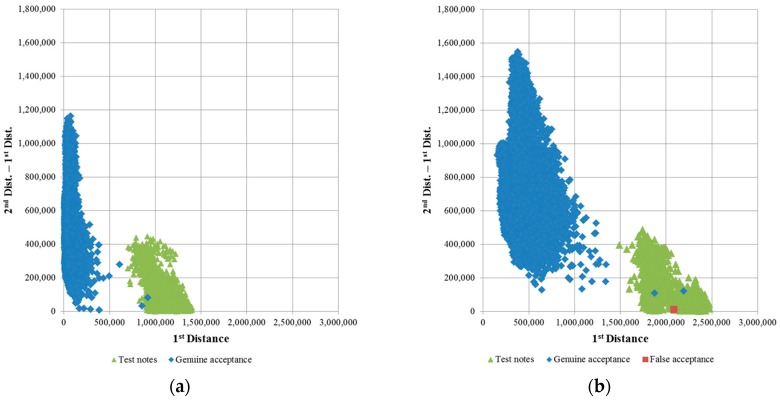
Scatter plots of matching distances of real USD banknotes and test notes obtained using 388 similarity map pixels and (**a**) 20 PCA dimensions; (**b**) 388 PCA dimensions.

**Figure 7:**
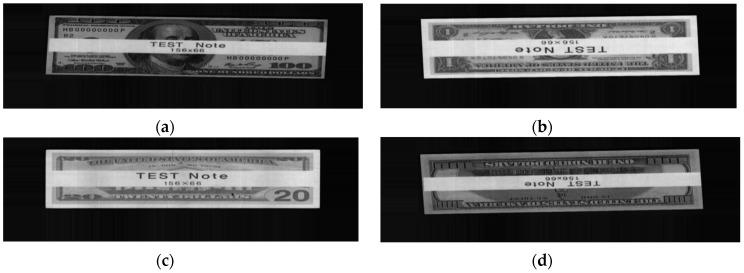
Examples of test notes (**a**) A direction; (**b**) B direction; (**c**) C direction; (**d**) D direction.

First, we draw a scatter plot of the 1st distances and the differences between the 1st and 2nd distances of the genuine acceptance cases of USD, as shown in Figure 6. The system must have the ability to reject the unrecognized cases. To simulate the rejected cases, we use the test notes on which the patterns were modified, as shown in Figure 7. Because the test notes are unrecognized, their distances to all the banknote class center vectors are greater than those of genuine banknotes. Therefore, their 1st distances are greater, and the positions of the test note cases on the matching distance scatter plot are far away from those of the genuine accepted banknote cases. Figure 6 shows an example of a scatter plot of matching distances of real USD banknotes and test notes.

It can be seen from Figure 6 that the matching score distributions of banknotes and test notes are separated in the plots and that the degree of varies with the dimensionality of the extracted PCA features. When applying a threshold for rejection based on matching distances, the test notes must be rejected and, consequently, the error cases and uncertain banknote cases (banknotes that are damaged, soiled, faded, *etc*.) will also be rejected. If the score distributions of genuine-acceptance banknotes and test notes are well separated, the uncertain and error cases are easier to reject, and the recognition results are more reliable. To evaluate the separation between these matching score distributions, we calculate the distributions’ scatter values based on distribution centers using Fisher’s criterion in LDA [23]. For each test, we obtain two distances *d_X_* and *d_Y_*, namely the 1st distance and the difference between the 1st and 2nd distance. The center of each distribution is at the position (*µ_X_*, *µ_Y_*). The measure of scatter of the matching distance distribution is equivalent to a variance and is calculated as follows:

(8) $$S=\sum_{i=0}^{N-1}\left[{\left(d_{Xi}-\mu_{X}\right)}^{2}+{\left(d_{Yi}-\mu_{Y}\right)}^{2}\right]$$

where *N* is the number of samples in the distribution. The scatter values of the accepted cases and rejected cases are denoted by *S_A_* and *S_R_*, respectively. Using the Fisher criterion in LDA, our goal is to find the optimal number of PCA dimensions for banknote feature extraction so that the following ratio (called the F-ratio) is maximized:

(9) $$F=\frac{S_{B}}{S_{W}}=\frac{{\left(\mu_{AX}-\mu_{RX}\right)}^{2}+{\left(\mu_{AY}-\mu_{RY}\right)}^{2}}{S_{A}-S_{R}}$$

where *S_B_* and *S_W_* are the between-class scatter and within-class scatter, respectively, and (*µ_AX_*, *µ_AY_*) and (*µ_RX_*, *µ_RY_*) are the centers of the acceptance and rejection distributions, respectively.

## 3. Experimental Results

In this study, we used a database consisting of 99,236 images captured from 49,618 USD banknotes on both sides. The images in the database include the four directions of 17 types of banknotes: $1, $2, $5, recent $5, most recent $5, $10, recent $10, most recent $10, $20, recent $20, most recent $20, $50, recent $50, most recent $50, $100, recent $100 and most recent $100. The number of images in each banknote class is shown in Table 2. In our experimental database of USD banknote images, both the number of images and the number of classes are comparatively larger than those in previous studies, as shown in Table 3.

First, we calculated the similarity map and applied half-histogram thresholding to obtain the mask for selecting the discriminative areas in a 64 × 12-pixel sub-sampled banknote image. Using the resulting binary mask shown in Figure 5, we selected 388 gray values of the pixels corresponding to the white areas of the mask from the sub-sampled image for banknote classification.

**Figure 8:**
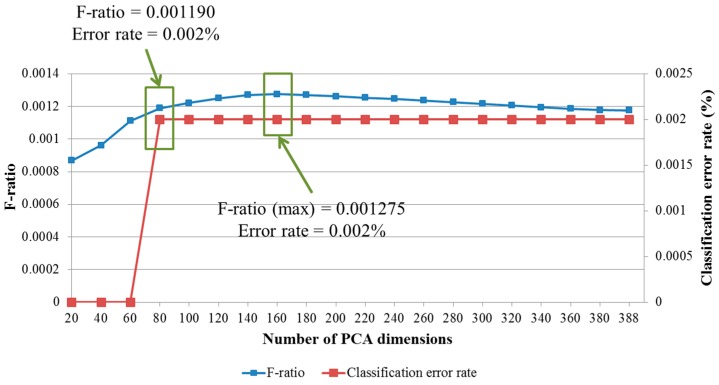
Example of average similarity map obtained from similarity maps of all USD classes and binary mask obtained from similarity map for feature selection.

From the selected banknote image data, we extracted the classification features using PCA. In this step, the reliability of the classification results is affected by the dimensionality of the extracted PCA feature vector, as explained in Section 2.4. Therefore, in subsequent experiments, we determined the optimal PCA dimensionality that yields the best classification accuracy and reliability in term of maximization of the F-ratio given by Equation (9). A USD test note database consisting of 2794 images was collected for our rejection test experiments. We considered test notes and false accepted cases of banknotes to belong to the same distribution, such that the remaining distribution consists of only genuine accepted cases. The experimental results for the F-ratio calculation and classification accuracy for various numbers of PCA dimensions are shown in Figure 8. The error rate was calculated based on unsupervised K-means clustering for 68 classes in the USD banknote database.

**Figure 9:**
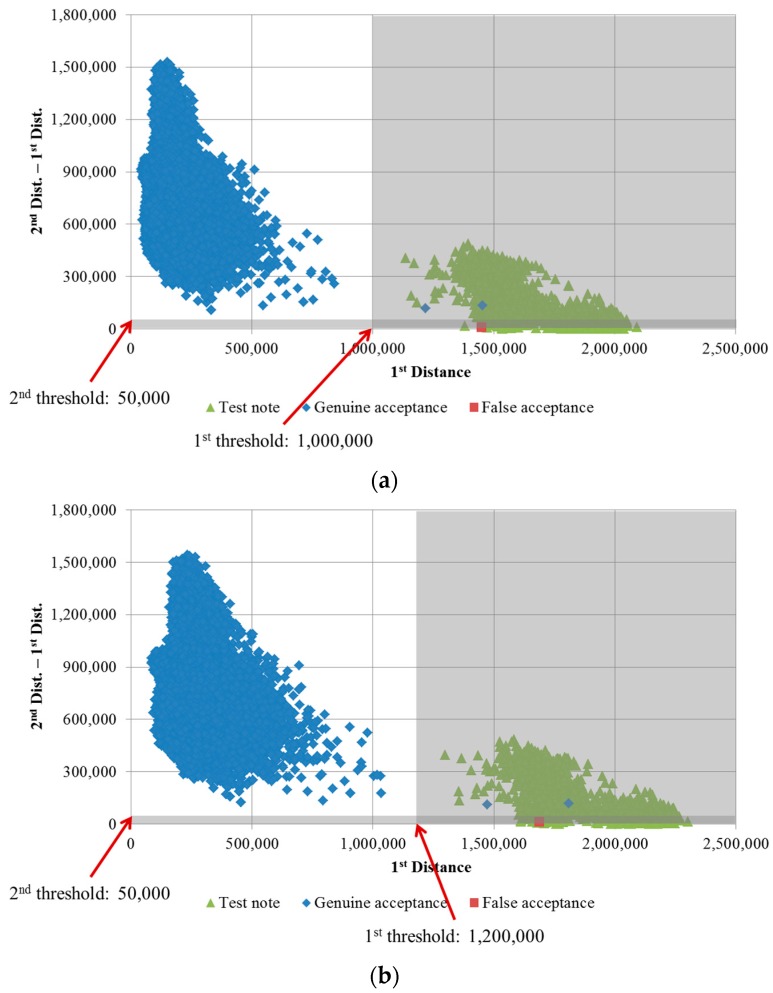
Scatter plot of matching distances of real USD banknotes and test notes using 388 similarity map pixels and (**a**) 80 PCA dimensions; (**b**) 160 PCA dimensions.

It can be seen from the Figure 8 that although there were no classification errors in the cases in which 20, 40, or 60 PCA dimensions were used for feature extraction, the separations between the distributions of genuine accepted cases and rejected cases were not good in terms of low ratios between each distribution’s scatter measures. The scatter plot of the matching distances when 20 PCA dimensions were used is shown in Figure 6a. The F-ratio reached a maximum value of 0.001275 at a dimensionality of 160. As the number of extracted features increases, much more processing time is required. Therefore, we considered two cases—the use of 80 or 160 PCA dimensions for feature extraction—in subsequent experiments conducted to evaluate the recognition accuracy achieved. Scatter plots of the distances for the cases of extraction of 80 and 160 PCA dimensions for banknote and test note matching tests are shown in Figure 9.

With the parameters for banknote feature extraction determined, we evaluated the accuracy of the proposed recognition method in comparison to the accuracy reported for methods used in previous studies, as shown in Table 4. When we used 80 or 160 PCA dimensions for feature extraction, there were no changes in the error rates and rejection rates, which were 0.002% and 0.004%. The rejected cases correspond to the banknotes for which the 1st matching distances were higher than the 1st threshold and for which the differences between the 1st and 2nd distances were lower than the 2nd threshold. The 2nd threshold is used to ensure that the recognition result is reliable, as explained in Section 2.4.2. In Figure 9, the positions of rejected cases on the scatter plots are in the gray areas. Because the total error and false rejection rate was 0.006%, the correct recognition rate of our method was 99.994%.

It can be seen from Table 4 that although the numbers of banknote images and classes in our USD database were greater than in other studies, the recognition accuracy of our method was higher than that in previous studies in terms of low false recognition rates and rejection rates. Consequently, we can confirm that our proposed method outperforms the previous previously proposed methods for USD banknote recognition.

**Figure 10:**
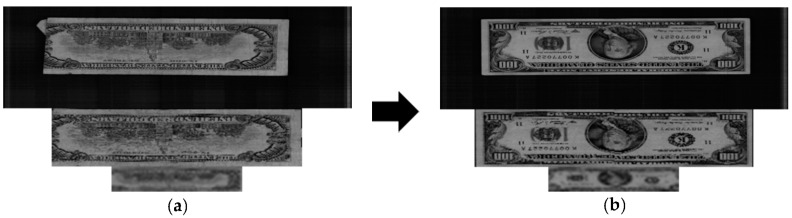
False recognition case of USD banknote: (**a**) Input banknote; (**b**) False recognized class.

The false recognition case for our method is shown in Figure 10, in which the uppermost image is the original banknote image and the middle and lower images are the deskewed image and the 64 × 12-pixel sub-sampled image of the upper image, respectively. The banknote on the left was misclassified as belonging to the class of the banknote on the right. This error case occurred because the image was captured from a folded banknote, as seen in Figure 10.

**Figure 11:**
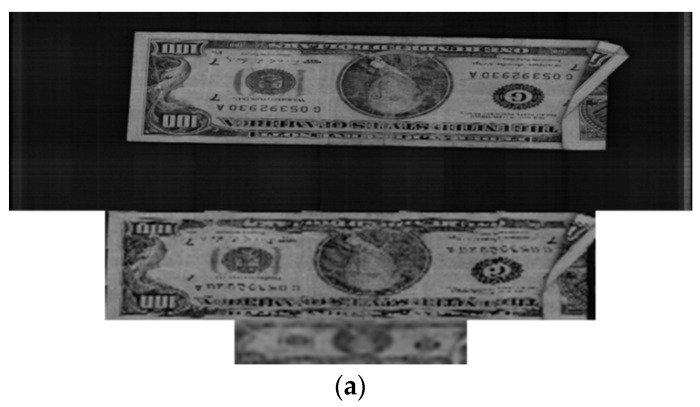

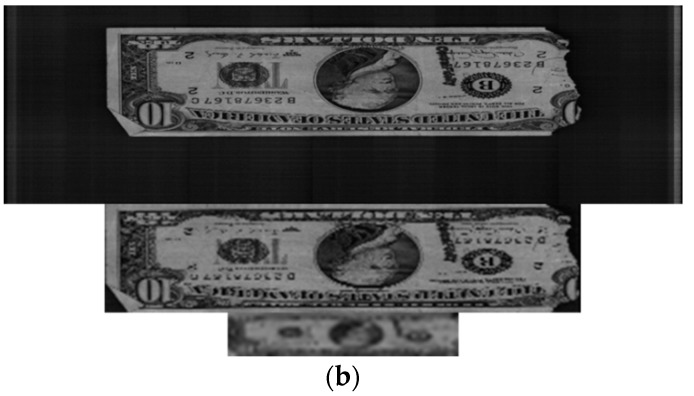
Rejection cases in USD banknote image database: (**a**) Case 1; (**b**) Case 2.

Figure 11 presents illustrations of rejection cases in our experiments in which the images are arranged in the same manner as in Figure 10. Although these input banknote images were correctly recognized, their matching scores were too high, so their distributions on the scatter plots in Figure 9 were in the rejection region, where the 1st distances are greater than the 1st threshold. It can be seen in Figure 11 that the upper case corresponded to images of a folded banknote, similar to the false recognition case in Figure 10. The remainder of the rejected images was captured from a severely damaged banknote with a tear, folded corner, and writing patterns. These resulted in 1st distances to the genuine classes of these banknote features being higher than the 1st rejection threshold and consequently the images being rejected by the system.

**Figure 12:**
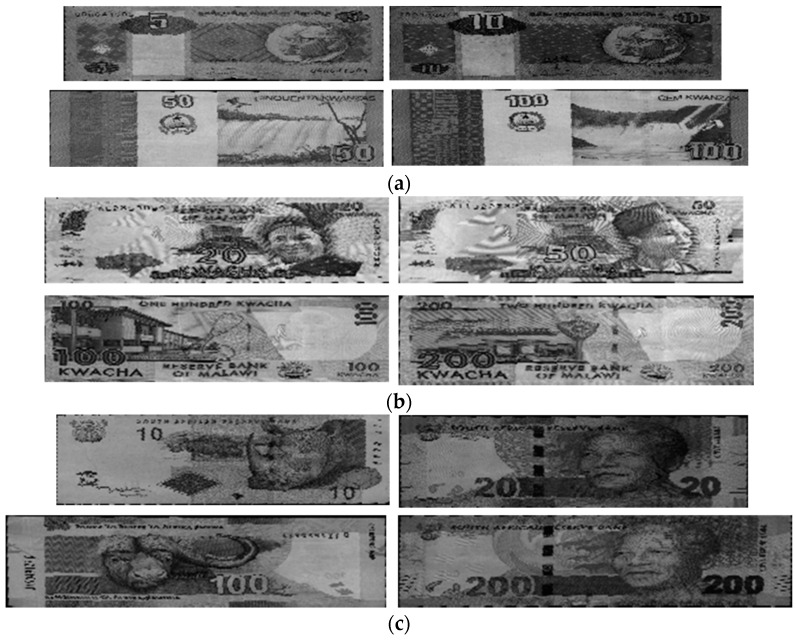
Examples of banknote images in the experimental databases: (**a**) Angolan kwanza (AOA); (**b**) Malawian kwacha (MWK); (**c**) South African rand (ZAR).

In subsequent experiments, we applied the proposed method to other countries’ banknote image databases to confirm the performance of our method for different types of paper currency. The banknotes used in these experiments were South African rand (ZAR), Angolan kwanza (AOA) and Malawian kwacha (MWK). The numbers of banknote images and classes in the experimental databases are shown in Table 5. Figure 12 shows some examples of banknote images for each type of currency.

Because test notes similar to those used in the USD experiments were not available for the AOA, MWK and ZAR currencies, we tested the recognition accuracy of these databases using the same parameters as those for USD recognition. In addition, because there has been no previous research on recognition of paper banknotes from Angola, Malawi, or South Africa, we were not able to compare the accuracy of our method with any methods applied to these currencies in previous studies. Our proposed method correctly recognized 100% of the banknote images in the AOA and ZAR databases and 99.675% of the banknotes in the MWK database. The experimental results for the similarity maps and the recognition error rates for the AOA, MWK and ZAR databases are given in Table 6.

From the images of the similarity maps and the resulting masks shown in Table 6, most of the selected areas for recognition in the MWK and ZAR banknote images were on the two sides. The recognition area in the AOA banknote images was located in the middle of the images. The reason for these results is that the patterns on these banknotes, such as numbers, seals, photos, and portraits, are asymmetrically distributed. In the cases of MWK and ZAR, the photo and portrait patterns are printed far to the two sides of the banknotes, while on AOA banknotes, the feature patterns are more different in the middle areas, depending on the banknotes’ denominations and directions. As a result, the high-discriminating-power regions for AOA, MWK, and ZAR banknote images were determined and are shown in Table 6.

**Figure 13:**
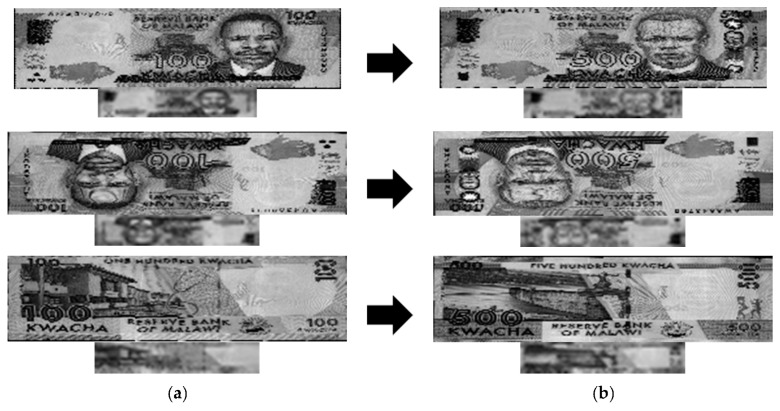
False recognition cases in MWK banknote image database: (**a**) Input banknote; (**b**) False recognized class.

Examples of error cases in the MWK database are shown in Figure 13. The lower image with smaller sizes for each pair was sub-sampled from the upper banknote image. In this figure, the MK100 banknote images of the front side and forward back side shown in the left column were misclassified as MK500 banknotes in the same direction, examples of which are shown in the right column. It can be seen from the Figure 13 that in the areas selected for recognition by map in Table 6, the shapes of the textures in the sub-sampled images of the MK100 and MK500 banknotes are slightly similar to each other. This resulted in misclassification in these cases. However, the banknote images in the AOA and ZAR databases were correctly recognized with error rates of 0%, so we can confirm that our proposed method can be applied to paper currencies from various countries and achieve good performance in terms of matching accuracy.

We also measured the processing time for our recognition method. This experiment was conducted using the USD banknote image database and a desktop personal computer (PC) with a 2.33 GHz CPU and 4 GB of memory. In these experiments, we compared the time required when using 80 and 160 PCA dimensions for the feature extraction process. When 160 PCA dimensions were used, the system was able to process approximately 442 images/s (1000/2.26 ms). When the number of extracted features by PCA was reduced by half, the processing speed increased to 568 images per second (1000/1.76 ms). Therefore, we used 80 PCA dimensions for final decisions using our banknote recognition method. The average processing times are shown in Table 7.

In a previous study [16] in which a higher-powered PC (3.5-GHz CPU, 8 GB of memory) was used, up to 5.83 ms was required to recognize an image. Our proposed method required less processing time for the following reasons. First, because we used deskewed banknote images of smaller sizes (up to 400 × 120 pixels, compared to 1212 × 246 in [16]), the sub-sampling time was reduced. Second, the number of extracted features used for recognition in our method was smaller (80 dimensions, compared to 192 in [16]). Therefore, the feature extraction and matching processes required less time than with the method used in [16].

In addition, we measured the processing time of a banknote-counting machine using a Texas Instruments (TI) digital media processor (chip). This machine required approximately 1 ms to process and recognize one input banknote using our proposed method with 80 PCA features extracted from a visible-light banknote image.

We also calculated the total memory usage required to employ our proposed method with a counting machine with limited resources. The measurement details are given in Table 8. The total memory usage was 931,924 Bytes for our method. Our proposed method outperformed the USD recognition method described in [16] in terms of both processing time (15.6 ms) and memory usage (1.6 MB).

## 4. Conclusions

In this paper, we propose an efficient banknote recognition method based on the selection of the high-discriminating-power regions of banknote images captured by visible-light sensors. The recognition regions are determined by calculating the ratio between the in-class similarity and between-class similarity of banknote images. Our experimental results show that using a PCA-based K-means algorithm as the classifier, our proposed method was able to recognize various paper currencies by denomination and input direction with high accuracy, as indicated by low error rates and rejection rates. With the help of a test note database that represents rejection cases, we were also able to evaluate the reliability of our method based on the measurement of scatter between genuine acceptance and rejection score distributions.

In future work, we plan to combine our recognition method with the evaluation of fitness for recirculation of banknotes to properly reject poor-quality banknotes that are difficult to recognize. In addition, we intend to extend our method for recognizing paper currencies from multiple countries, rather than recognition of denominations and directions of banknotes only from an individual country.

## Figures and Tables

**Table 1:** Comparison of proposed and previous methods.

Category	Method	Strength	Weakness
Using the whole banknote image	Using average brightness values of eight uniform rectangles on banknote images as the input for BP network [1]. Edge characteristic of linear transformed banknote image was used as the input for three layer NN classifier [2,3]. Using HMM to model textures of banknote as a random process [6]. Using GGD to extract statistical features from QWT coefficients [4].	Simple in feature extraction method [1]. Make use of all of the available recognition features on banknote image.	Only focused on orientation recognition of a banknote type—Renminbi (RMB) 100 Yuan [1]. Possibility of redundancy in the input data to the classifiers. Need for additional feature extraction or representation method because of large-size images could reduce classification speed (HMM [6], QWT [4]).
Using local regions on banknote image	Using symmetric masks on banknote images to select input features for the NN classifiers [7,8]. Optimizing masks for selecting features using GA algorithm [10]. Using SURF based on class-specific components of textures on banknote images [11]. Determination of discriminant areas on banknotes by multi-template correlation matching [12].	Help to reduce the size of input data to the classifier and reduce processing time. High-discriminating-power regions on banknote images could be located [10,11,12].	Fixed recognition regions on banknote images were not the optimal discriminative areas [7,8]. Difficulty in application of embedded systems with limited resources due to usage of complex features (SURF [11]).
Using statistical analysis to extract features from banknote image	Using PCA on data acquired by various sensors and LVQ for classification [13,14]. Applying PCA for feature extraction from banknote data acquired by various sensors: IR, UV, magnetic, fluorescence [15]. Using PCA for feature extraction, SVM for pre-classification, and K-means for denomination recognition [16]. Using CA on features extracted by wavelet transform [17].	Help to reduce the size of input data to the classifier. Can be applied to feature extraction from data acquired by multiple sensors [13,14,15].	Additional processing time and resources required for feature extraction by statistical analysis (memory for PCA eigenvector data).
Combining two feature extraction methods: local region definition and statistical analysis	ROIs were selected from five security features on Indian banknote image. PCA was used for dimensionality reduction of data extracted from ROI [18]. Using LDA for feature reduction on ROIs containing textures cropped from Indian banknote image [19]. Using PCA for feature extraction from the region on the right part of detected banknote image [20]. Using feature extraction method based on PCA on banknote areas selected by similarity map **(proposed method)**.	Input feature to the classifiers was reduced in dimensionality and optimized by statistical analysis.	Using large-size scanned color banknote images that are difficult to apply on embedded systems [18]. ROI selection had to be conducted with the help of external tool (Mazda [19]). The selected region for recognition on the right part of banknote image is not definitely optimal [20]. Calculation of similarity map is necessary **(proposed method)**.

**Table 2:** Numbers of banknote images in experimental USD database.

Type of Banknote	A Direction	B Direction	C Direction	D Direction
$1	2018	2016	2018	2016
$2	1626	1660	1626	1660
$5	849	834	849	834
Recent $5	1208	1218	1208	1218
Most Recent $5	1795	1797	1795	1797
$10	1498	1509	1498	1509
Recent $10	1258	1277	1258	1277
Most Recent $10	1564	1565	1564	1565
$20	1651	1647	1651	1647
Recent $20	1063	1069	1063	1069
Most Recent $20	1965	1959	1965	1959
$50	1270	1262	1270	1262
Recent $50	1397	1343	1397	1343
Most Recent $50	1479	1573	1479	1573
$100	1011	1126	1011	1126
Recent $100	1964	1761	1964	1761
Most Recent $100	1250	1136	1250	1136

**Table 3:** Numbers of images and classes in the experimental databases used in previous studies and in this study.

Study	Number of Images	Number of Classes
[4]	15,000	24
[13]	3600	24
[14]	3570	24
[16]	61,240	64
[25]	65,700	48
This study	99,236	68

**Table 4:** Comparison of recognition accuracy of the proposed method and previous studies.

Recognition Method	Experimental USD Banknote Image Database	Error Rate (%)	Rejection Rate (%)
[4]	15,000 images/24 classes	0.120	0.580
[16]	61,240 images/64 classes	0.114	0.000
Proposed method	99,236 images/68 classes	0.002	0.004

**Table 5:** Numbers of banknote images and classes in the experimental databases of Angolan kwanza (AOA), Malawian kwacha (MWK) and South African rand (ZAR).

Currency	Number of Images	Number of Classes
AOA	1366	36
MWK	2464	24
ZAR	760	40

**Table 6:** Experimental results for the AOA, MWK, and ZAR banknote image databases.

Currency	Similarity Map	Binary Mask	Error Rate (%)
AOA	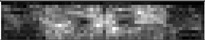	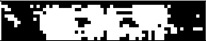	0.000
MWK	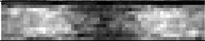	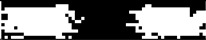	0.325
ZAR	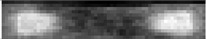	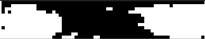	0.000

**Table 7:** Processing time of the proposed recognition method on desktop computer (unit: ms).

Number of PCA Dimensions	Sub-Sampling	Feature Extraction	K-Means Matching	Total Processing Time
160	1.23	0.78	0.25	2.26
80 (Proposed)	1.23	0.40	0.13	**1.76**

**Table 8:** Calculation of memory usage of our proposed method.

Category	Data Size	Data Type	Memory Usage (Bytes)
Original image	1584 × 464	BYTE	734,976
Deskewed image	400 × 120	BYTE	48,000
Sub-sampled image	64 × 12	BYTE	768
Similarity map	388	Integer	1552
Selected region by similarity map	388	BYTE	388
PCA transform matrix	80 × 388	Integer	124,160
Extracted PCA features	80	Integer	320
K-means centers	80 × 68	Integer	21,760
**Total**			931,924
